# Adeno-Associated Virus-Mediated Rescue of the Cognitive Defects in a Mouse Model for Angelman Syndrome

**DOI:** 10.1371/journal.pone.0027221

**Published:** 2011-12-09

**Authors:** Jennifer L. Daily, Kevin Nash, Umesh Jinwal, Todd Golde, Justin Rogers, Melinda M. Peters, Rebecca D. Burdine, Chad Dickey, Jessica L. Banko, Edwin J. Weeber

**Affiliations:** 1 Department of Molecular Pharmacology and Physiology, University of South Florida, Tampa, Florida, United States of America; 2 Department of Pharmacy, University of South Florida, Tampa, Florida, United States of America; 3 Department of Neuroscience, Princeton University, Princeton, New Jersey, United States of America; 4 University of South Florida Health Byrd Alzheimer's Institute, Tampa, Florida, United States of America; 5 McKnight Brain Institute, University of Florida, Gainesville, Florida, United States of America; 6 Center for Translational Research in Neurodegenerative Diseases, University of Florida, Gainesville, Florida, United States of America; 7 Department of Molecular Medicine, University of South Florida, Tampa, Florida, United States of America; National Institutes of Health/NICHD, United States of America

## Abstract

Angelman syndrome (AS), a genetic disorder occurring in approximately one in every 15,000 births, is characterized by severe mental retardation, seizures, difficulty speaking and ataxia. The gene responsible for AS was discovered to be *UBE3A* and encodes for E6-AP, an ubiquitin ligase. A unique feature of this gene is that it undergoes maternal imprinting in a neuron-specific manner. In the majority of AS cases, there is a mutation or deletion in the maternally inherited *UBE3A* gene, although other cases are the result of uniparental disomy or mismethylation of the maternal gene. While most human disorders characterized by severe mental retardation involve abnormalities in brain structure, no gross anatomical changes are associated with AS. However, we have determined that abnormal calcium/calmodulin-dependent protein kinase II (CaMKII) regulation is seen in the maternal *UBE3A* deletion AS mouse model and is responsible for the major phenotypes. Specifically, there is an increased αCaMKII phosphorylation at the autophosphorylation sites Thr^286^ and Thr^305/306^, resulting in an overall decrease in CaMKII activity. CaMKII is not produced until after birth, indicating that the deficits associated with AS are not the result of developmental abnormalities. The present studies are focused on exploring the potential to rescue the learning and memory deficits in the adult AS mouse model through the use of an adeno-associated virus (AAV) vector to increase neuronal *UBE3A* expression. These studies show that increasing the levels of E6-AP in the brain using an exogenous vector can improve the cognitive deficits associated with AS. Specifically, the associative learning deficit was ameliorated in the treated AS mice compared to the control AS mice, indicating that therapeutic intervention may be possible in older AS patients.

## Introduction

Angelman syndrome is a genetic disorder resulting in severe mental retardation, ataxic gait, lack of speech, seizures that may be difficult to control, and frequent laughter [1]. The gene responsible for AS is *UBE3A* and it is unique in that it is one of a small family of human imprinted genes. *UBE3A* undergoes maternal imprinting in the brain; thus, the paternal copy is silenced and the only active copy inherited is maternal. AS can occur from a variety of genetic abnormalities of the 15q11–q13 chromosome, each of which render the *UBE3A* gene silenced. The majority of AS cases (70%) occur through a *de novo* deletion (∼4 Mb) of 15q11–q13 of the maternal chromosome which incorporates the *UBE3A* gene [2], but it can also occur as a result of abnormal methylation of the maternal copy, preventing its expression [3], [4] or uniparental disomy in which two copies of the paternal gene are inherited [5], [6]. The remaining AS cases arise through various *UBE3A* mutations of the maternal chromosome or they are diagnosed without a genetic cause (12–15%). Current estimates indicate that AS is present in about one in every 15,000 births [7], though the actual number of diagnosed AS cases is lower likely due to misdiagnosis. *UBE3A* codes for the E6-associated protein (E6-AP) ubiquitin ligase. E6-AP is an E3 ubiquitin ligase, therefore it exhibits specificity for its protein targets, which include the tumor suppressor molecule p53 [8], a human homologue to the yeast DNA repair protein Rad23 [9], E6-AP itself, and Arc, the most recently identified target [10], [11]. The anatomy of the mouse and human AS brain shows no major alterations compared to the normal brain, indicating the cognitive deficits may be biochemical in nature as opposed to developmental [12], [13].

The AS mouse model used in this study, which contains a disruption of the maternal *UBE3A* gene through a null mutation of exon 2, was developed by Jiang et al. (1998). This model has been incredibly beneficial to the field of AS research due to its ability in recapitulating the major phenotypes characteristic of AS patients. For example, the AS mouse has inducible seizures, poor motor coordination, hippocampal-dependent learning deficits, and defects in hippocampal LTP. We previously found that the cognitive deficits in the AS mouse model are associated with abnormalities in the phosphorylation state of calcium/calmodulin-dependent protein kinase II (CaMKII) [14]. There was a significant increase in phosphorylation at both the activating Thr^286^ site as well as the inhibitory Thr^305^ site of αCaMKII without any changes in total enzyme level, resulting in an overall decrease in its activity. There was also a reduction in the total amount of CaMKII at the postsynaptic density, indicating a reduction in the amount of active CaMKII. With this identification, a mutant mouse model was developed with a point mutation at the Thr^305^ site preventing phosphorylation. When this mutant CaMKII mouse model was crossed with the AS mouse, the phenotype of the AS mouse was rescued. Seizure activity, motor coordination, hippocampal-dependent learning, and LTP were restored similar to wildtype levels. These studies were the first to identify a particular defect in AS associated with the resulting AS phenotype. The preponderance of αCaMKII is expressed postnatally in the mammalian brain suggesting that the major phenotypes of the AS mouse model are due to postnatal biochemical alterations as opposed to a global developmental defect [15]. However, because this study utilized a mutation in CaMKII, the possibility that improvements were due to developmental rescue could not be formally excluded.

In this study, we sought to determine if the major hippocampal-dependent cognitive defects could be rescued in the adult mouse through a *UBE3A* gene replacement strategy in the hippocampus using recombinant adeno-associated virus (rAAV) serotype 9, which has been shown to have one of the highest transgene expressions in the brain compared to other serotypes. The vector used in this study is a type 2 terminal repeat (TR2)-flanked *UBE3A* gene, and the control vector is a TR2-flanked green fluorescent protein (GFP). We found that by using an exogenous *UBE3A* gene administered directly into the hippocampus, there was a significant improvement in associative learning. This is the first study demonstrating cognitive rescue in adult AS mice and indicates that therapeutic intervention in AS patients may be efficacious even into adulthood.

## Results

**Figure 1:**
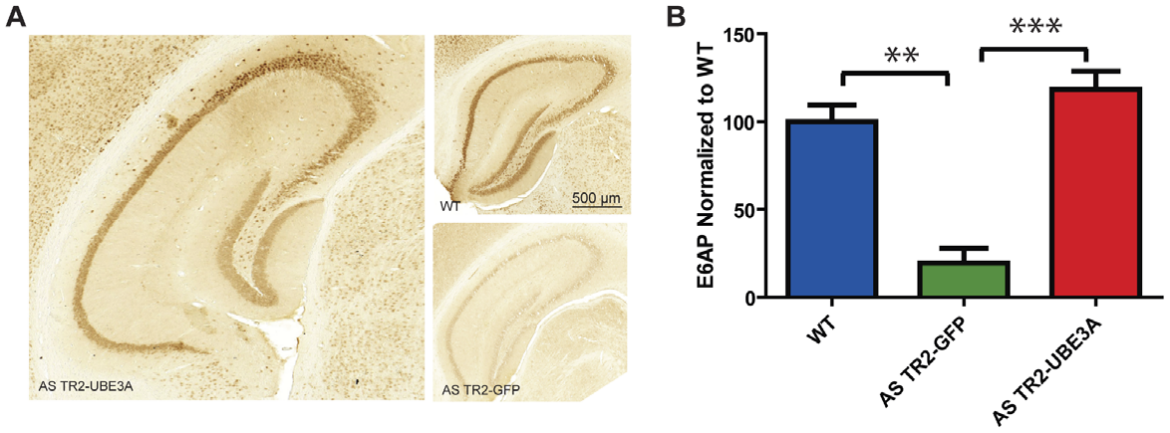
E6-AP protein levels were restored to wildtype levels in the TR2-UBE3A treated AS mice. (A) Representative coronal slices through the hippocampus from each group stained for E6-AP. (B) Quantitative analysis of the IHC revealed a significant increase in E6-AP expression in the WT and AS-TR2-UBE3A mice compared to the AS TR2-GFP group, while there was no significant change between the WT and AS TR2-UBE3A mice. Results shown represent the mean with standard error.

As previously mentioned, one of the targets of E6-AP is itself. This is hypothesized to be a mechanism to regulate levels of maternally expressed protein; however, we were concerned that transfected neurons would express significantly more E6-AP protein than normally observed from WT maternal gene expression. Quantitative assessment of neuronal E6-AP was determined by immunohistochemistry 8 weeks following transduction (Fig. 1A). Quantified immunoreactivity of E6-AP determined for the entire hippocampal formation revealed equal amounts of E6-AP expression in AS TR2-UBE3A mice compared to wild type (WT) age matched controls and barely detectable in AS TR2-GFP mice (Fig. 1B ANOVA Tukey [F(2,154) = 9.422, *P<0.0001*]). These results show that AS TR2-UBE3A mice do not overexpress E6-AP protein and have levels comparable to wild type mice.

**Figure 2:**
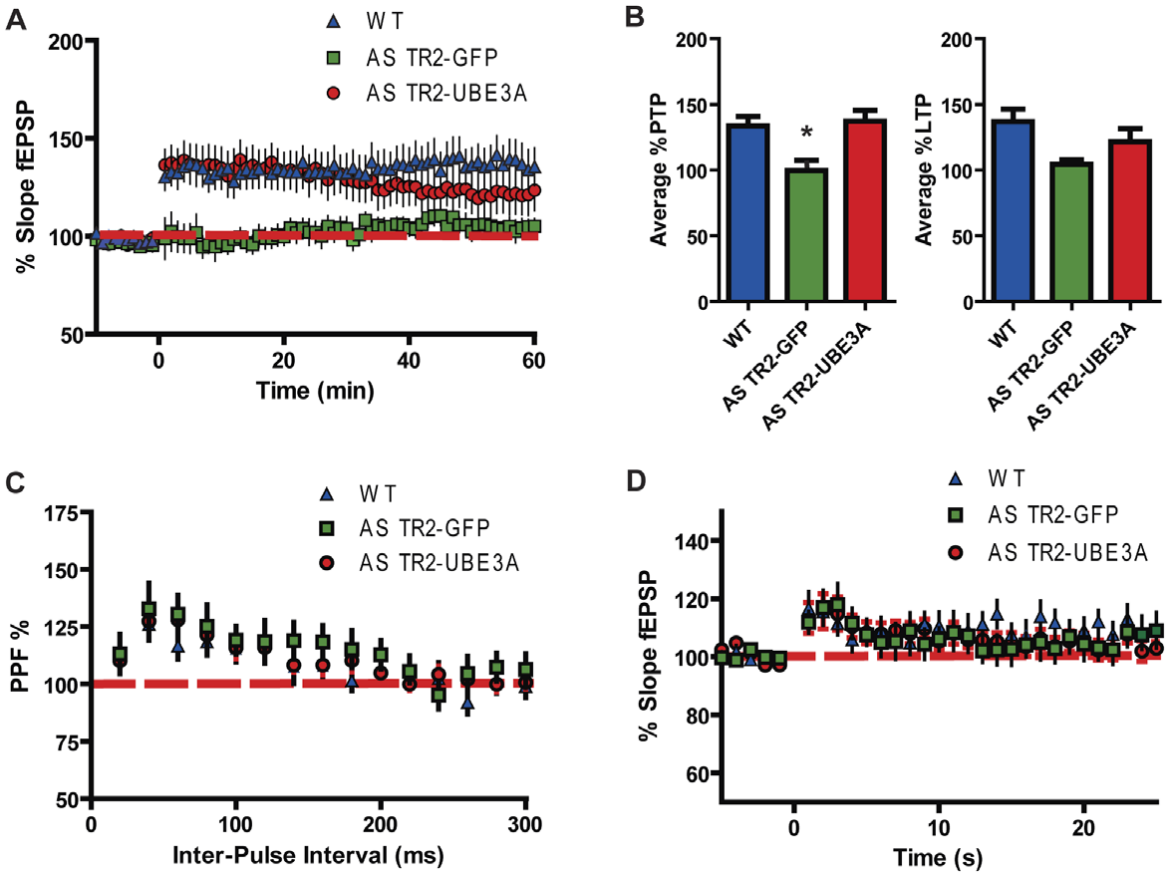
Increasing E6-AP in the AS mouse results in improvements in early phase LTP. (A) AS TR2-GFP mice have significant deficits in hippocampal synaptic plasticity. LTP was induced following 20 min of baseline recordings. (B) Immediately following TBS, acute hippocampal slices taken from AS TR2-GFP mice had significant deficits in the average PTP (average of first 5 min recordings of fEPSPs slopes). To compare late phase LTP, the last 5 min recordings of fEPSPs slopes were averaged, and there was no significant difference between any of the groups. (C) There were no significant differences between any of the groups in either PPF or (D) PTP, indicating that short term synaptic plasticity mechanisms are unaffected. Results shown represent the mean with standard error.

A severe deficit in LTP induction is well established in the AS mouse model [12]. We measured LTP in area CA1 from hippocampal slices of AS TR2-UBE3A and AS TR2-GFP mice and compared this to WT responses. LTP was induced using a theta-burst stimulation (TBS) protocol as this high frequency stimulation (hfs) protocol reveals the greatest difference in LTP induction between AS and WT mice [14]. Consistent with previous reports, AS TR2-GFP mice showed a severe LTP deficit compared with WT mice (Fig. 2A); however, the post-tetanic potentiation (PTP) and early phase of LTP in AS TR2-UBE3A mice was equivalent to WT (Fig. 2B ANOVA Tukey [F(2,44) = 6.017, *P<0.05*]). There was no significant difference seen between any of the groups during late phase LTP, which compared the last 5 minutes of fEPSPs slopes (Fig. 2B ANOVA Tukey [F(2,43) = 2.865, *P>0.05*]). There was a slow decline in potentiation ∼30 minutes post-stimulation. This level was maintained throughout the experiment to 60 minutes, suggesting that *UBE3A* expression can partially rescue the LTP defect in the AS mouse model using a non-saturating hfs. There was no change in presynaptic function determined by pre-pulse facilitation (PPF) (Fig. 2C) and the rescue in early phase LTP induction was not the result of enhanced PTP (Fig. 2D).

**Figure 3:**
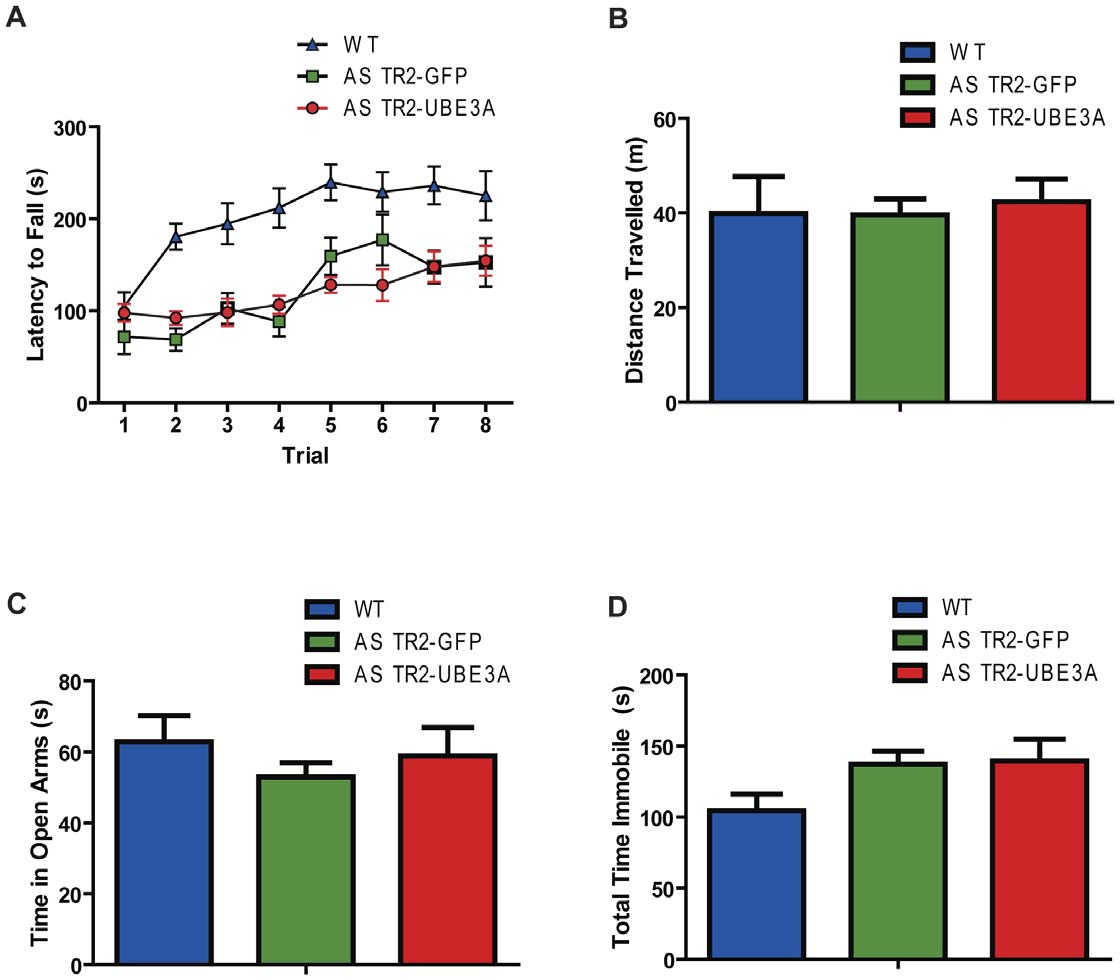
There were no changes in motor coordination, activity levels, or anxiety. (A) There was no change in latency to fall of the rotorod in either AS group. (B) Total distance travelled during the open field test revealed no significant difference between any of the treatment groups. (C) Time spent in the open arms of the elevated plus maze was used to determine general anxiety. (D) Time spent immobile in the elevated plus maze was not significantly different in any of the groups. Results shown represent the mean with standard error.

The AS mouse model exhibits significant defects in motor coordination [12], [16], [17]. A single injection of rAAV in the hippocampus did not significantly increase *UBE3A* expression in the cerebellum (data not shown), and we found that rotorod performance in AS TR2-UBE3A mice is not changed compared to AS TR2-GFP controls (Fig. 3A). To ensure that the motor coordination defect in the AS TR2-UBE3A mice was not the result of altered activity or anxiety, an open field (Fig. 3B ANOVA Tukey [F(2,27) = 0.07563, *P>0.05*)]) and elevated plus maze test (Fig. 3C ANOVA Tukey [F(2,27) = 0.5290, *P>0.05*] and Fig. 3D ANOVA Tukey [F(2,27) = 2.472, *P>0.05*]) were performed and showed no changes in AS TR2-UBE3A versus AS TR2-GFP mice.

**Figure 4:**
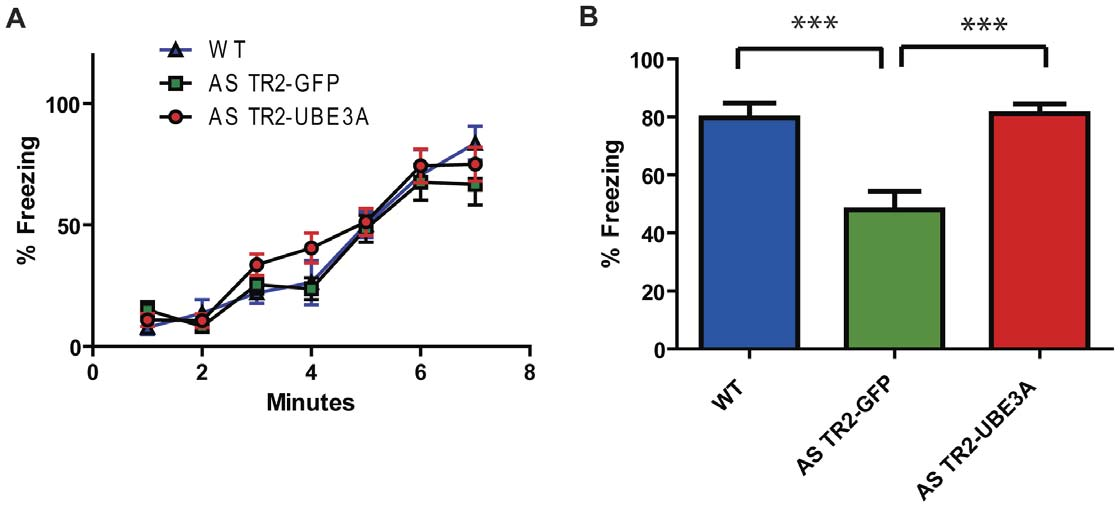
AS mice receiving TR2-UBE3A had significant improvements in associative learning. (A) There were no differences during the training phase of fear conditioning, indicating that all groups of mice were capable of freezing to the same extent. (B) AS TR2-GFP mice show significant deficits in contextual fear conditioning when assessed 24 h after training. AS TR2-UBE3A mice, however, froze at the same rate as the wildtype mice. Results shown represent the mean with standard error.

Associative fear conditioned learning is disrupted in the AS mouse model [12], [16]. Contextual fear conditioned learning was assessed through freezing behavior to the context 24 hours after training. Although there was no change between any of the groups during the training phase of fear conditioning (Fig. 4A), our AS TR2-UBE3A mice show associative learning comparable to that of WT mice (Fig. 4B ANOVA Tukey [F(2,55) = 71.65, *P<0.0001*]) when placed back in the same context 24 hours after training. There were no significant differences seen in the cued test between any of the groups 24 hours after training (data not shown).

**Figure 5:**
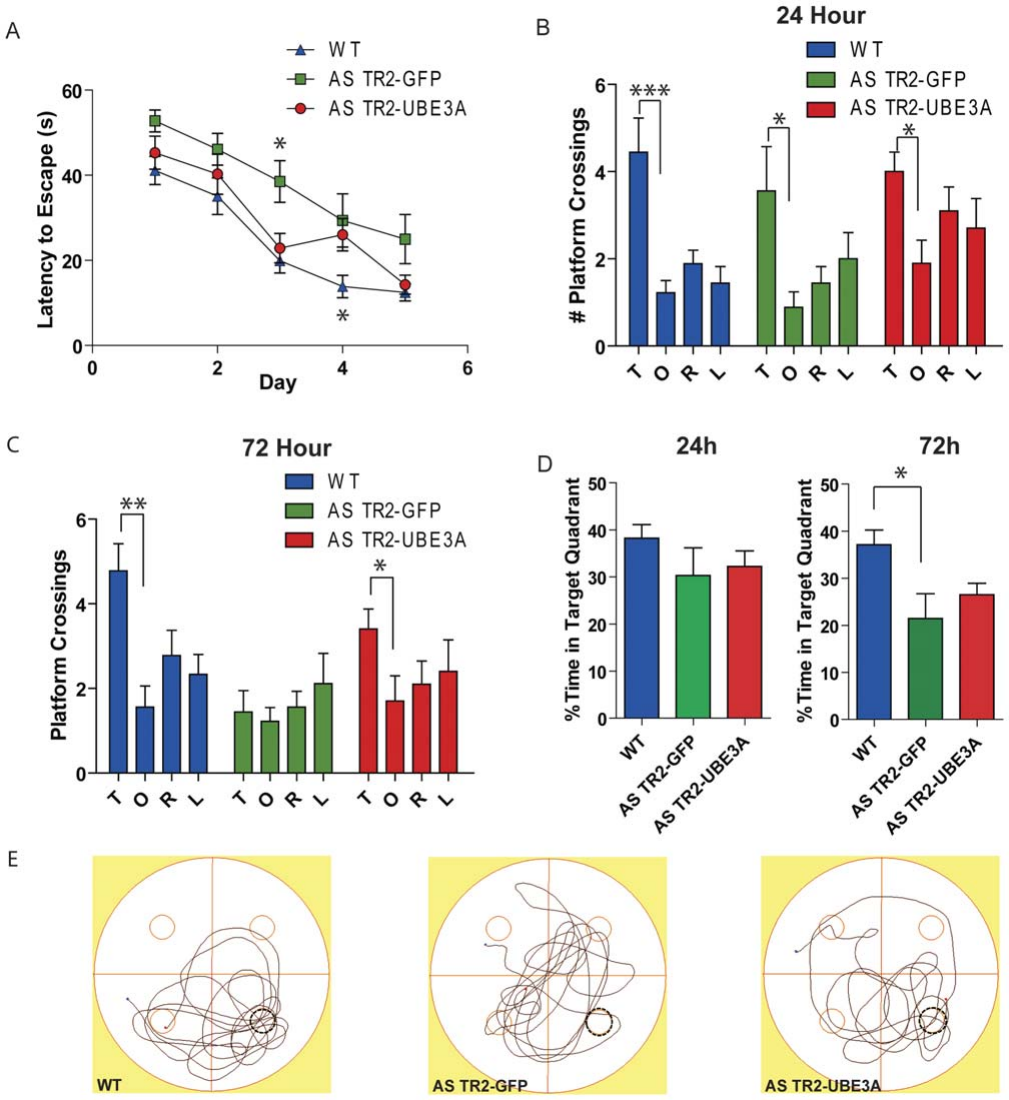
AS TR2-UBE3A mice had significant improvements in the Morris water maze. (A) Escape latency to reach the platform during 5 days of training in Morris water maze. The only significant differences seen were an increase in latency for the AS TR2-GFP mice on day 3 and a decrease in latency for wildtype mice on day 4 compared to the other two groups (2-way ANOVA Bonferroni: Interaction [F(8,100) = 1.01, *P>0.05*]; Treatment [F(2,100) = 5.30, *P<0.05*]; Time [F(4,100) = 53.49, *P<0.0001*]; Matching [F(25,100) = 5.37, *P<0.0001*]). The target platform is indicated by the black circles. (B) Quantification of the number of platform crossings in the target (T), opposite (O), right (R), and left (L) quadrants during the probe trial of the Morris water maze 24 hours after training indicate no significant differences among any of the groups in comparing target platform crossings to opposite platform crossings (ANOVA Tukey WT: [F(3,35) = 9.546, *P<0.0005*]; AS TR2-GFP: [F(3,35) = 3.186, *P<0.01*]; AS TR2-UBE3A: [F(3,39) = 2.814, *P<0.05*]). All three groups learned the platform location based on a spatial bias as indicated by the time spent in the target quadrants (Fig. 5D 24 h ANOVA Tukey [F(2,26) = 1.027, *P>0.05*]) (C) A probe test 72 hours after training indicate that the WT and AS TR2-UBE3A groups had significantly more target platform crossings compared to the number of opposite platform crossings (ANOVA Tukey WT: [F(3,35) = 6.086, *P<0.005*]; AS TR2-GFP: [F(3,35) = 0.5650, *P>0.05*]; AS TR2-UBE3A: [F(3,39) = 2.679, *P<0.05*]), but this improvement was not spatially biased as seen by the time spent in the target quadrant (Fig. 5D 72 h ANOVA Tukey [F(2,25) = 5.067, *P<0.02*]). Results shown represent the mean with standard error.

Another form of learning shown to be disrupted in the AS mouse model is spatial learning evaluated using the Morris water maze task. All groups were trained for 5 days to find the hidden platform and recorded latencies find that AS TR2-UBE3A have significantly lower latency compared to AS TR2-GFP mice on training days 3 and 4 (Fig. 5B). A probe trial given 24 hours after 5 days of training showed a significant difference in the number of target platform crossings compared to opposite platform crossings in all three groups (Fig. 5B). There was also no significant difference in the time spent in the target quadrant between groups (Fig. 5D) suggesting equivalent normal spatial memory formation for all groups. However, when tested 72 hours after the last day of training (day 5), the WT and AS TR2-UBE3A mice had significantly more target platform crossings compared to opposite platform crossings (Fig. 5C). Despite the increase in platform crossings seen with the TR2-UBE3A mice, this is not associated with a spatial bias to the target quadrant (Figure 5D,E). Neither the TR2-UBE3A nor AS TR2-GFP showed a significant increase for the target quadrant above chance (25%) or compared to WT mice. Thus, AS TR2-UBE3A mice retained a search strategy for the target platform without a significant spatial bias to the target quadrant. The difference observed between AS TR2-GFP and AS TR2-UBE3A mice is not a result of increased activity, greater swim speed or decreased anxiety.

## Discussion

Recent clinical trials for disorders categorized as developmental, such as Fragile X mental retardation and Rett syndrome, are showing promising results for cognitive disruption and associated behavioral symptoms [18], [19], [20], [21], [22]. Angelman syndrome has biochemical and genetic associations with both of these disorders [23], [24], [25]. We previously showed a genetic rescue of the major phenotypes in the AS mouse model using a specific mutation to alpha CaMKII [16]. Temporal regulation of alpha CaMKII expression results in little expression prior to post natal day 5 followed by increased expression to maximal levels by post natal day 21 [26]. The lack of appreciable CaMKII expression during development of the central nervous system suggested that CaMKII's effect was altering synaptic function resulting in the phenotype rescue. The present study sought to determine if replacement of E6-AP protein in the hippocampal formation was sufficient to recover the memory and synaptic plasticity defects associated with hippocampus dysfunction in the adult AS mouse model.

The use of the AAV-9 serotype particle allowed for a preponderance of neurons within the hippocampus and entorhinal cortex to show E6-AP protein accumulation. While the LTP defect was not completely recovered, we were able to show a recovery of the associative learning and memory defect when compared to controls. Memory was also affected in the AS TR2-UBE3A mice seen as an increase in non-spatial biased platform crossing 72 hours following training. These results suggest that the increase in hippocampal LTP is associated with the amelioration of defects in the Morris water maze and associative learning and memory. Not surprising was the remaining defect in motor coordination. This defect has been considered to reside in the cerebellum, where we found no detectable E6-AP production. However, these results provide the first evidence that the cognitive disruption associated with Angelman syndrome is not a developmental defect and can be reversed in the adult AS mouse model. Moreover, these data support the possibility of developing a beneficial therapeutic to treat the cognitive and motor defects seen in adult human Angelman syndrome.

## Materials and Methods

### Ethics Statement

All animal testing procedures were approved by the Institutional Animal Care and Use Committee of the University of South Florida and followed the NIH guidelines for the care and use of laboratory animals (Approval ID number A4100-01).

### Vector Construction

The *UBE3A* plasmid M43 clone (NCBI database U82122) was a gift from Yong-Hui Jiang. *UBE3A* was sub-cloned into the pTR12.1-MCS vector (which contains the short hybrid CMV chicken beta-actin promoter as described in Mah et al. [27] at the *Spe I* and *Cla I* cloning sites and sequenced. Virus was generated by cotransfection of the *UBE3A* plasmid with the helper plasmids pXX6 and pAAV9 in HEK293 cells (ATCC, Manassas, VA). The resulting recombinant virus was purified using an iodixanol gradient as previously described [28]. A dot-blot assay was used to determine the viral titer and is expressed as vector genomes (vg)/ml.

### Breeding of animals

Mice with the *UBE3A* null mutation (AS) were described previously [12]. All experiments were performed on mice that have been backcrossed to the 129/SvEv line (Jackson Labs, Bar Harbor, ME) at least 5 generations. Female mice containing the null mutation were bred with 129/SvEv (WT) males to produce maternally-deficient AS offspring and WT littermate controls. Animals were kept on a 12 hour light/dark cycle and food and water provided *ad libitum*. All animal testing procedures were approved by the Institutional Animal Care and Use Committee of the University of South Florida and followed the NIH guidelines for the care and use of laboratory animals.

### Intrahippocampal AAV Injections in AS Mice

Mice are anesthetized with isoflurane and placed in the stereotaxic apparatus (51725D Digital Just for Mice Stereotaxic Instrument, Stoelting, Wood Dale, IL). An incision is made sagitally over the middle of the cranium and the surrounding skin is pushed back to enlarge the opening. The following coordinates are used to locate the left and right hippocampus: AP −2.7 mm, L±2.7 mm, and V −3.0 mm. Mice received bilateral intrahippocampal injections of either TR2-UBE3A particles at a concentration of 1.5×10^12^ genomes/mL (N = 10) or TR2-GFP particles at a concentration of 1.4×10^12^ genomes/mL (N = 9) using a 10 µL Hamilton syringe. Recombinant viral vectors in 1 µL volume were co-administered with 1 µL of 20% mannitol in each hemisphere. The wound is cleaned with saline and then closed using Vetbond (NC9286393 Fisher Scientific, Pittsburgh, PA). Mice recovered in a clean, empty cage on a warm heating pad and were then singly housed until sacrificed.

### General Activity and Anxiety

For all subsequent behavior testing, the following number of animals were used for each group: 9 WT, 9 AS TR2-GFP, 10 AS TR2-UBE3A. Activity was measured by the open field test. Mice were placed in a 40×40 cm acrylic chamber under normal lighting conditions and allowed to explore for 15 min. Video tracking software monitored movement, immobility, and distance traveled (ANY-Maze, Stoelting, Wood Dale, IL). Anxiety was measured by the elevated plus maze (EPM). The EPM consisted of two well-lit open arms (35 cm) and two enclosed arms (35 cm) facing each other. Each arm was attached to a common center platform (4.5 cm) and the entire device was elevated 40 cm off the ground. Each mouse was placed in the center platform and allowed to explore for 5 min. Immobility was measured after the mouse remained motionless for a minimum of 2 consecutive sec.

### Motor coordination

The accelerating rotorod was used to assess motor coordination and motor learning (Ugo Basile, Italy). Mice were placed on a 3 cm diameter rod with an initial rotation of 4 rpm and accelerated to 40 rpm over a maximum of 5 min. Mice were tested for latency to fall off the rod for 4 trials over 2 consecutive days.

### Associative fear conditioning

Fear conditioning was used to assess hippocampus function and memory formation. Mice were placed in a 25×25 cm sound-attenuation chamber with a wire grid floor. Mice were allowed to explore the context for 2 min before they received the conditioned stimulus (CS, 90 db tone) for 30 sec. At the end of the 30 sec, mice received a mild foot shock (0.5 mA: unconditioned stimulus (US)). After 1.5 min, the mice received a second CS/US pairing and monitoring continued for 2.5 min. Freezing was assessed by the use of a weight transducer system (Panlab, Spain). Mice were considered freezing if movement ceased for at least 2 consecutive sec. 24 hrs following CS/US presentation, mice were placed back into the chamber and allowed to explore for 3 min.

### Spatial memory

The Morris Water maze test was used to determine spatial memory formation. A 1.2 m diameter pool was filled with white opaque water. A 10 cm diameter white platform was submerged just below the water surface and large extra-maze cues positioned around the room. Mice were placed in the pool and allowed to swim to the escape platform for a maximum of 60 sec. Mice were given 4 trials per day for 5 days. Latency to escape and swim speed was measured by video tracking software (ANY-Maze, Stoelting, Illinois). 24 and 72 hours following the 5^th^ day of training, the platform was removed and swim patterns were monitored for 60 sec.

### Hippocampal slice preparation and extracellular recording

One hemisphere of each brain was used for electrophysiology and the remaining hemisphere was preserved for histology. Slice preparations and the theta-burst stimulation LTP protocol used were previously described by Weeber et al. [29]. Briefly, the theta burst stimulation consists of five trains of four pulses at 100 Hz with an interburst interval (IBI) of 20 seconds.

### Histology

Each hemisphere that was not used for electrophysiology was immersion fixed in 4% PFA. Prior to sectioning with the cryostat, tissue was transferred to 30% sucrose overnight. Endogenous peroxidases were quenched with 0.3% hydrogen peroxide for 30 min before the blocking solution (2% BSA, 5% goat serum, 0.25% Triton-X) was applied for one hour. Tissue sections were then incubated overnight with antibody solution (2% BSA, 0.25% Triton-X) with E6AP (A300-352A Bethyl Laboratories, Inc.) at 1∶300. After washing with PBS, secondary antibody (Goat Anti-Rabbit IgG(H+L) SouthernBiotech) was applied for one hour at room temperature. Immunoreactivity was detected using a metal-enhanced DAB substrate kit (Pierce). A Mirax Micro digital slide scanner (Carl Zeiss USA) was used to photograph IHC sections and MIRAX SCAN software was used to quantify E6-AP expression in the hippocampus. Expression levels were normalized to the wildtype.
